# The Function of Osteopathic Medicine in the Treatment of Adhesive Capsulitis

**DOI:** 10.7759/cureus.27640

**Published:** 2022-08-03

**Authors:** Leonid Tafler, Abbey Santanello, Yelizaveta Lysakova

**Affiliations:** 1 Family Medicine, Touro College of Osteopathic Medicine, New York City, USA; 2 Medicine, St. George’s University, New York City, USA

**Keywords:** osteopathic medicine, family medicine, omm research, osteopathic manipulative medicine (omm), adhesive capsulitis

## Abstract

Adhesive capsulitis (frozen shoulder) may result from any injury to the shoulder. The main signs and symptoms are pain, decreased range of motion, and stiffness of the joint. A patient could have additional causes that can include muscle spasm, rotator cuff tear, or weakness of the upper extremities, which could make treatment decisions more complicated. Multiple therapeutic and surgical approaches exist. Successful outcomes for adhesive capsulitis are due to timely diagnosis and effective treatment options.

In our case report, a 58-year-old female developed acute sharp pain in her shoulders, decreased range of motion, as well as bilateral weakness in her upper extremities. The symptoms developed after doing repetitive overhead arm motion while swimming without proper conditioning. The pain was debilitating and prevented the patient from completing simple daily maneuvers. Significant weakness in the upper extremities made it impossible for her to independently dress herself.

Initially, the patient's symptoms were attributed to the chronic effects of osteoarthritis and rotator cuff tear. One orthopedic surgeon recommended replacement of the right shoulder. As a second opinion, another orthopedic surgeon recommended bilateral shoulder replacement. The orthopedic surgeons' decisions were based on physical examination and reading the MRI, which confirmed total rotator cuff tear and osteoarthritis. Before proceeding with surgical treatment, the patient was evaluated by an osteopathic specialist.

The osteopathic specialist's decision was based on osteopathic principles that the body is a unit capable of self-regulation and that structure and function are reciprocally interrelated. Observation and palpation are particularly important means of uncovering information. After the first examination, the osteopathic physician diagnosed and successfully restored cervical spine mobility problems (cervical somatic dysfunctions) and relieved a trapezius muscle spasm. The patient's upper extremities gained strength after the first treatment and she was able to dress herself (which is a task she was unable to do in over a year with continuous physical therapy treatment).

At this point, surgical treatment became an alternative option. The patient preferred to continue osteopathic manipulation and osteopathic manipulation under anesthesia. The patient experienced a resolution of her symptoms over time. A stepwise approach to management is necessary for patient assessment and diagnosis, especially when the alternative recommendation is surgery.

## Introduction

Adhesive capsulitis (also known as frozen shoulder syndrome) may result from any injury of the shoulder including other comorbidities such as diabetes. The main symptoms and signs are pain, decreased range of motion, and stiffness of the joint. In frozen shoulder, adhesions make the shoulder capsule thick, which prevents normal motion. The patient could have additional symptoms such as muscle spasms and weakness of the upper extremities, which could make the treatment plan more complicated [1].

The traditional treatment for frozen shoulder is a stepwise approach with the use of non-steroidal anti-inflammatory drugs, steroid injections, physical therapy, and finally surgery. The use and efficacy of osteopathic manipulative medicine (OMM) and OMM under anesthesia in a stepwise fashion have not been well-reported in the treatment of frozen shoulder. Osteopathic manipulation under anesthesia (OMA) is a very effective therapeutic modality, using safe and proven osteopathic techniques and strategies. The technique is typically used in cases where more conventional strategies failed to produce results.

Leonid Tafler, doctor of osteopathic medicine (DO) and the first author of this article, is a leader in osteopathic manipulation under anesthesia having performed over 2,000 osteopathic manipulation procedures with propofol. This allows the osteopath to stretch and manipulate muscle tissue and joints without the patient’s involuntary movement resisting the motion. When the patient is sedated with propofol, they can still feel pain in the affected joint. It is a very valuable feature and helps to avoid complications. As a result, this manipulation technique can be an even safer alternative to standard OMM.

Propofol has two advantages: muscle relaxation and additionally the ability of the patient to still feel pain, visualized by facial expression, which allows the physician to determine the extent of range of motion during manipulation. This allows the physician to safely perform the procedure.

Here, we describe the case of a different diagnostic and therapeutic approach for frozen shoulder management incorporating the use of osteopathic manipulative medicine for initial management.

## Case presentation

A 58-year-old female, without past medical history, presented with a one-year history of bilateral shoulder pain, stiffness, decreased range of motion, and complicated by severe weakness of the upper extremities. The symptoms developed after doing a repetitive overhead arm motion while swimming without proper conditioning. About 10 minutes into her swim, she felt a sudden sharp and severe pain in both shoulders and upper back. Immediately following the pain, came bilateral upper limb weakness, which had rendered her incapable of swimming back to shore without assistance.

The initial sharp pain had subsided and developed into a constant dull pain, which resulted in substantial inconvenience. The pain was debilitating and prevented the patient from completing simple daily maneuvers. Significant weakness in the upper extremities made it impossible for her to independently dress herself. Prior to the injury, the patient lived an active lifestyle and would exercise every day. Her typical exercise regime included walking and resistance-based arm exercises, including the use of resistance bands and 10-pound weights.

She visited an orthopedic surgeon and detailed the events of her acute presentation. Examination and radiology reports suggested severe arthropathy of the joint. The surgeon ultimately recommended a left shoulder arthroplasty. Seeking a second opinion, another orthopedic surgeon recommended bilateral shoulder replacement.

After careful evaluation of the risks and benefits of the procedure, she decided to explore other conservative options. At this time, she began going to physical therapy on a weekly basis and completed several weeks of treatment. The patient had noted some slight improvement in her pain, however, sufficient improvement in her mobility, bilateral weakness, and upper limb strength was not optimally achieved.

Thirteen months after the inciting event, the patient sought out an osteopathic specialist for reassessment and management. Physical examination revealed (1) diffuse upper extremity pain with guarding, (2) limited bilateral pan-directional passive and active range of motion at the shoulder joint (flexion/abduction 60-degree bilaterally, internal rotation-sacral level bilaterally), and (3) decreased grip-strength in both hands (2/6 bilaterally). By taking the history of presentation, and the clinical findings of physical examination into account, a diagnosis of adhesive capsulitis was postulated [2].

**Figure 1:**
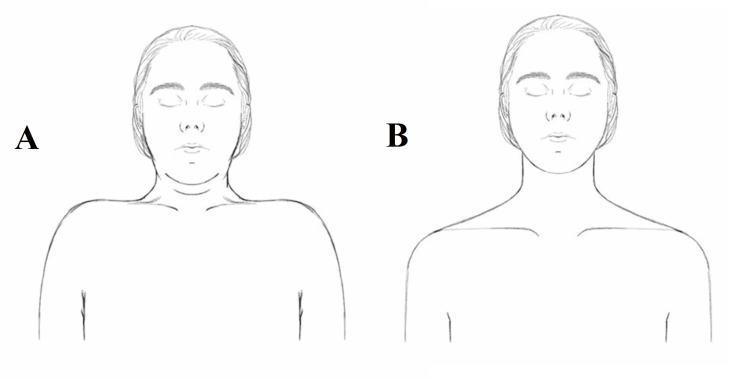
Comparison of bilateral trapezius spasm with a normal presentation. The images show (A; left) the patient has a bilateral trapezius spasm restricting the patient’s range of motion and (B; right) normal, absence of shoulder spasm.

Classic osteopathic examination using the tenderness, asymmetry, restricted motion, tissue texture changes (TART) approach, multiple cervical and thoracic pathological changes and severe chronic trapezius muscle spasm (Figure 1A) were noted compared to a normal presentation (Figure 1B). High-velocity low amplitude technique was performed on the cervical and thoracic region. Following her first session of osteopathic manipulative medicine, the patient reported immediate relief of hand weakness, significant alleviation of pain by more than 50%, and improved shoulder range of motion. This improvement supports the osteopathic philosophy that structure governs function.

After two months of conventional OMM, the shoulders’ range of motion failed to progress, so the physician performed osteopathic manipulation under anesthesia. Using modified spencer technique, shoulder range of motion was improved up to 90% of normal range.

Since the initiation of treatment, she has continued to follow up at the clinic for two years every two to three months. At the present moment, she reports that she is freely able to complete most tasks involving her upper limbs, without pain or the need for assistance. She is able to carry on with daily activities that include household chores, and cooking. This marks a dramatic improvement from her initial presentation, in which she was unable to even lift lightweight items or dress herself. She notes that she occasionally requires assistance with overhead activities, such as reaching the top shelf of the kitchen cabinet. Even remarkably so, she was able to return to her previous exercise regime, occasionally participating in stretching and strength exercises to improve her mobility without surgical treatment [3].

## Discussion

Adhesive capsulitis is a common phenomenon and is not difficult to diagnose. In our case, the patient had preexisting conditions, such as osteoarthritis and bilateral torn rotator cuffs, confirmed by MRI imaging. The chronic muscle spasm and cervical spine derangement, not an MRI imaging, was the key to successful diagnosis of the adhesive capsulitis, but it was neglected or misdiagnosed.

In most chronic cases, diagnostic imaging is for supportive documentation only and to assist in selecting safe and effective osteopathic techniques. The goal of osteopathic manipulative medicine is a treatment of somatic dysfunctions, which are pathological responses to the musculoskeletal system due to improper functioning [4]. Over a century, this osteopathic therapeutic modality has been developed as primary or adjunctive therapy that aims to restore function and homeostasis to an affected area and the body as a whole.

The use of anesthesia in conjunction with traditional osteopathic techniques was a reasonable alternative to invasive surgery. OMM under anesthesia has been in practice since the 1930s by orthopedic surgeons and osteopathic physicians as an alternative to failed conservative methods and surgical intervention [4]. Propofol is a common anesthetic agent used to relax skeletal muscle by acting on the voltage-gated sodium channels in muscle spindles [4]. With complete relaxation of the muscle reflexes and pain fibers, larger articulatory motions can be performed to lyse fibrotic and scarred tissue caused by chronic inflammation [4].

Each medical specialty and subspecialty have advantages and limitations which require collaboration of specialists and a stepwise approach to the therapy. According to Table 1, the osteopathic approach to treatment is compared to other specialists [5-7]. The advantage of an osteopathic physician is the application of osteopathic philosophy, assessment, and unique techniques.

**Table 1:** Comparison between the approaches of osteopathic physicians vs. other specialists.

	Osteopathic approach	Traditional approach
Initial assessment	Comprehensive history and physical; osteopathic assessment using palpatory skills, special tests, ROM testing, and TART findings	Comprehensive history and physical; ROM testing
Diagnosis	Osteopathic assessment and palpation skills, MRI as supporting documentation	MRI for primary documentation
Treatment	Osteopathic manipulation, manipulation under anesthesia, medication	Surgery, medication, physical therapy
Recovery	Immediate improvement possible, ability to immediately assess increased ROM and strength testing	Possible hospitalization, risk of complications, surgery recovery longer due to complexity of operation, near to long term improvement possible
Long term management	May require repeat treatments for weeks or months to reach optimal healing	May require physical therapy and additional support after surgery; risk of infection and/or failure of joint depending on type of surgery

## Conclusions

Adhesive capsulitis is a common phenomenon that occurs as a result of increased inflammation, eventually leading to the fibrosis of the glenohumeral joint. Conventional therapy with non-steroidal anti-inflammatory drugs, physical therapy and corticosteroid injection may not be effective in chronic settings. Surgery could be excessive, non-effective or create post-surgical complications, such as developing more scar tissue. When patients are offered surgical intervention, it is beneficial to consult with an osteopathic specialist for possible alternatives. Osteopathic manipulation enhanced with the use of anesthesia is safe, effective and alternative to surgical intervention technique.
